# Beyond the tumour microenvironment: Harnessing border immunity for glioblastoma immunotherapy

**DOI:** 10.1002/ctm2.70841

**Published:** 2026-09-18

**Authors:** Lanjie Chen, Yixin Gao, Jian Zhong, Nu Zhang

**Affiliations:** ^1^ Department of Neurosurgery The First Affiliated Hospital of Sun Yat‐sen University Guangzhou China; ^2^ Department of Neurosurgery Guangdong Provincial People's Hospital (Guangdong Academy of Medical Sciences) Southern Medical University Guangzhou China; ^3^ Medical Research Institute Guangdong Provincial People's Hospital (Guangdong Academy of Medical Sciences) Southern Medical University Guangzhou China

Glioblastoma (GBM) is one of the most aggressive primary tumours of the central nervous system (CNS). Despite multimodal treatment including surgery, radiotherapy, and chemotherapy, the median survival of patients with GBM remains approximately 15 months.^1^ Immunotherapy has emerged as one of the most promising therapeutic strategies in oncology and has achieved remarkable success in several solid tumours, including lung cancer and melanoma.^2^, ^3^ However, GBM remains largely refractory to current immunotherapeutic approaches. Historically, efforts to improve GBM immunotherapy have primarily focused on tumour cells and the tumour microenvironment (TME).^4^ Unlike other solid tumours, however, the CNS possesses unique anatomical and immunological characteristics, including the blood‐brain barrier (BBB) and the absence of conventional lymphatic drainage within the brain parenchyma, which collectively contribute to a relatively restricted immune environment and impose spatial limitations on the recruitment and activity of peripheral immune cells within the TME.^5^, ^6^ These limitations raise the possibility that improving GBM immunotherapy may require consideration of immune regulatory compartments beyond the tumour itself.

CNS border structures are specialised anatomical interfaces located between the CNS and the peripheral environment, including the meninges, the choroid plexus, the skull bone marrow, and the perivascular spaces. Recent advances in CNS immunology have challenged the traditional view of the brain as an exclusively immune‐privileged organ, revealing that these border compartments serve as important interfaces connecting CNS immunity with peripheral immunity.^6^ Among these structures, the dura mater has emerged as one of the most prominent immune interfaces due to its extensive vascular network, meningeal lymphatic vessels, and specialised immune cell populations.^6^, ^7^

Recently, Zhang and colleagues reported a study that revealed the critical role of meningeal blood vessel blockage (MBB) in restricting GBM progression.^8^ The authors demonstrated that MBB remodels the dural microenvironment (DME), enhances anti‐tumour immune responses, improves the efficacy of GBM immunotherapy, and ultimately restrains tumour growth. By targeting the DME, MBB provides a promising translational strategy for neuro‐oncology. Importantly, this study shifts the current paradigm of GBM immunity from a tumour‐centric TME model towards a broader dura‐cerebrospinal fluid (CSF)‐TME immune axis, expanding our understanding of the spatial organisation of anti‐tumour immunity in GBM.

The mechanism underlying MBB‐mediated suppression of GBM progression can be summarised as follows. MBB restricts the entry of circulation‐derived border‐associated macrophages (cBAMs) into the dura and induces a CSF‐1‐rich DME, thereby promoting the expansion of resident BAMs (rBAMs). These FcRn‐high rBAMs display robust antigen‐presenting capacity, enabling efficient presentation of tumour‐derived antigens from the CSF. This cascade promotes T cell activation, amplifies anti‐tumour immune responses, and ultimately restricts GBM progression. MBB reshapes the composition and balance of macrophage subsets, favouring the expansion of a functionally superior immune population. These findings indicate an important principle of CNS border immunity: enhancing immune function may rely more on precisely remodelling cellular composition rather than simply increasing immune cell numbers.

The restricted accessibility of peripheral immune cells to the CNS renders alternative immune reservoirs at CNS borders attractive targets for therapeutic intervention. The dura mater, with its unique anatomical and immunological properties, represents an underappreciated immune compartment capable of supporting anti‐tumour immunity. The team proposed MBB as an innovative strategy to modulate meningeal blood supply and therefore target the DME. As a surgical intervention, MBB has a direct clinical correlate in humans—middle meningeal artery embolisation (MMAE). As an established procedure, MMAE has already demonstrated safety and efficacy in neurological disorders such as chronic subdural hematoma.^9^ Consequently, integrating MMAE as an immunomodulatory adjunct into existing multi‐modal treatment paradigms would not require an entirely new therapeutic framework, underscoring its translational feasibility and expanding the functional scope of neurosurgical interventions.

Based on the mechanism, MMAE may provide several potential benefits for GBM patients. First, it could serve as an immune‐priming strategy to enhance the efficacy of existing immunotherapies, such as immune checkpoint blockade, particularly in tumours characterised by insufficient antigen presentation or limited immune activation. By improving the local immune context, MMAE may enhance the response to immunotherapy. Second, compared with preclinical models, the availability of bilateral MMAE embolisation in humans may enable more precise and effective modulation of meningeal blood supply, enabling targeted intervention of the meningeal immune compartment with improved safety and procedural feasibility. Finally, as a localised intervention, MBB is expected to cause limited systemic toxicity relative to conventional pharmacological therapies, representing a comparatively safe strategy for GBM patients.

Although MBB represents a promising therapeutic concept, further studies are required to define its translational potential. Future investigations should determine the optimal therapeutic window, appropriate timing and clinical settings for MBB intervention, approaches for evaluating treatment outcomes and its potential combination with existing immunotherapeutic strategies. In addition, deeper characterisation of rBAMs may facilitate the development of more precise strategies to harness this immune compartment without requiring vascular intervention.

Collectively, by targeting the dura, MBB represents a surgical strategy for potentiating GBM immunotherapy with clinical translational potential. Future studies will be needed to validate its safety, therapeutic efficacy, and compatibility with current GBM treatment regimens, which will be essential for advancing its clinical translation.

## AUTHOR CONTRIBUTIONS

Lanjie Chen drafted the manuscript. Yixin Gao, Jian Zhong and Nu Zhang revised the manuscript. All authors reviewed and approved the final version of the manuscript. Ethics statement is not applicable. This article does not involve any human participants or animal subjects.

## CONFLICT OF INTEREST STATEMENT

The authors declare no conflict of interest.
